# Myocardial blood flow under general anaesthesia with sevoflurane in type 2 diabetic patients: a pilot study

**DOI:** 10.1186/1475-2840-13-62

**Published:** 2014-03-23

**Authors:** Carolien SE Bulte, Charissa E van den Brom, Stephan A Loer, Christa Boer, R Arthur Bouwman

**Affiliations:** 1 Department of Anaesthesiology, Institute for Cardiovascular Research, VU University Medical Center, De Boelelaan 1117, 1081 HV Amsterdam, The Netherlands; 2 Laboratory for Physiology, Institute for Cardiovascular Research, VU University Medical Center, Amsterdam, The Netherlands

**Keywords:** Type 2 diabetes mellitus, Myocardial blood flow, Anaesthesia, Hyperaemia

## Abstract

**Background:**

In type 2 diabetic patients, cardiac events in the perioperative period may be associated with diminished myocardial vasomotor function and endothelial dysfunction. The influence of sevoflurane anaesthesia on myocardial endothelial dysfunction in type 2 diabetic mellitus is investigated in this pilot study.

**Methods:**

Six males with type 2 diabetes mellitus and eight healthy controls were included. Using myocardial contrast echocardiography, myocardial blood flow (MBF) was measured at rest, during adenosine-induced hyperaemia (endothelium-independent vasodilation) and after sympathetic stimulation by the cold pressor test (endothelium-dependent vasodilation). Measurements were performed before and after induction of sevoflurane anaesthesia.

**Results:**

Sevoflurane anaesthesia decreased resting MBF in diabetics but not in controls (P = 0.03), while baseline MBF did not differ between diabetics and controls. Without anaesthesia, adenosine-induced hyperaemia increased MBF in both groups compared to resting values. Adenosine combined with sevoflurane resulted in a lower hyperaemic MBF in both groups compared to no anaesthesia. Differences in MBF in response to adenosine before and after sevoflurane administration were larger in diabetic patients, however not statistically significant in this pilot group (P = 0.08). Myocardial blood flow parameters after the cold pressor test were not different between groups.

**Conclusion:**

These pilot data in type 2 diabetic patients show that sevoflurane anaesthesia decreases resting myocardial blood flow compared to healthy controls. Further, we observed a trend towards a lower endothelium-independent vasodilation capacity in diabetic patients under sevoflurane anaesthesia. Endothelium-dependent vasodilation was not affected by sevoflurane in diabetic patients. These data provide preliminary insight into myocardial responses in type 2 diabetic patients under general anaesthesia.

**Trial registration:**

http://www.clinicialtrials.gov, NCT00866801

## Background

Diabetes mellitus is a major risk factor for cardiovascular morbidity and mortality [1]. In the perioperative period, cardiovascular events such as myocardial ischemia are more likely to occur in patients with diabetes mellitus [2]. These perioperative cardiac events may be associated with abnormalities in myocardial blood flow (MBF) due to endothelial dysfunction, an entity characterized by an impaired ability of the resistance vessels to dilate, or an enhanced response to vasoconstrictor agonists [3,4]. Hyperglycaemia is a major contributing factor in the development of endothelial dysfunction in type 2 diabetes [5,6]. Furthermore, impaired insulin signalling in endothelial cells, dyslipidaemia and altered secretions of adipokines from adipose tissue also have a detrimental role on vascular function [7].

Myocardial endothelial function can be evaluated by measuring vasodilator responses to sympathetic stimulation (primarily endothelium-dependent vasodilation) and to vascular smooth muscle relaxing agents, such as adenosine and dipyridamole (primarily endothelium-independent vasodilation). Studies in humans with type 2 diabetes have shown attenuation of myocardial responses to adenosine and dipyridamole [8-10]. Also, progressive worsening of the endothelium-dependent vasodilator capacity of the myocardium with increasing insulin-resistance was demonstrated in type 2 diabetic patients [8]. Myocardial responses to sympathetic stimulation were even further diminished in patients with an associated cardiac autonomic neuropathy [9].

Sevoflurane is a frequently used volatile anaesthetic with coronary vasodilating properties [11,12]. Experimental studies showed preservation of myocardial perfusion during sevoflurane anaesthesia if perfusion pressure was maintained [13-15]. Also, a study in isolated rat hearts reported a decreased vasodilator response to adenosine during sevoflurane administration [16]. Recently, we showed that sevoflurane anaesthesia did not affect resting MBF in cardiovascular healthy subjects. The vasodilator response to adenosine (endothelium-independent vasodilation) was diminished compared to measurement without anaesthesia. Endothelium-dependent vasodilation was not affected by sevoflurane. Human and experimental data on MBF and endothelial function under general anaesthesia in type 2 diabetes mellitus are lacking.

We hypothesised that myocardial endothelial function is affected by general anaesthesia in patients with type 2 diabetes mellitus. This pilot study was undertaken to investigate how type 2 diabetic patients respond to myocardial vasodilators under sevoflurane anaesthesia when compared to healthy controls.

## Methods

### Participants

The local Human Subjects Ethics Committee of the VU University Medical Center in Amsterdam, the Netherlands, approved this study (ID 2008/304). Subsequent patients were included after written informed consent. We prospectively studied 6 patients with type 2 (non-insulin dependent) diabetes mellitus and 8 healthy control subjects, all scheduled for non-cardiac surgery under general anaesthesia. Six out of 8 healthy volunteers were selected based on sex from a previously published study by Bulte *et al.*[17]. The diabetic subjects had haemoglobin A1c levels of 6.8 ± 0.8% (range 5.5 – 7.6%) and fasting glucose levels of 8.5 ± 1.5 mmol/l (range 6.7 – 10.9 mmol/l). Mean duration of type 2 diabetes was 7.7 ± 5 year (range 3 – 15 years), diabetic patients only received oral antidiabetic therapy. Exclusion criteria for participation included age < 18 years, allergy to echocardiographic contrast agents or adenosine, previous history of coronary artery disease, chronic obstructive pulmonary disease and use of beta-adrenergic blocking agents.

### Study protocol

All participants made an extra visit to our hospital for screening, autonomic function testing and baseline myocardial blood flow measurements. On the day of surgery the MBF measurements were repeated after the induction of sevoflurane anaesthesia but before the start of the surgical procedure.

### Myocardial blood flow measurements

As previously described, transthoracic myocardial contrast echocardiography (MCE) was performed using an iE33 ultrasound scanner equipped with a S5 – 1 transducer (Philips Medical Systems, Best, The Netherlands) [18]. A contrast agent consisting of microbubbles filled with sulphur hexafluoride with a mean diameter of 2,5 μm was used (Sonovue, Bracco Imaging, Milan, Italy) and continuously infused using a specific syringe pump (VuEject, Bracco SA, Switzerland). After two minutes of microbubble infusion, baseline perfusion images were acquired from apical 4-, 2- and 3-chamber views. Subsequently, hyperaemia was induced by a continuous infusion of 0.14 mg kg^-1^ min^-1^ adenosine. Finally, sympathetic stimulation was triggered by the cold pressor test (CPT) by immersing the hand of the patient in ice water (2 – 4°C) for 3 minutes. Perfusion sequences were recorded and consisted of 10 cardiac cycles of low acoustic power (mechanical index [MI] 0.17) imaging for microbubble detection followed by a burst of high acoustic power (MI 0.64) for microbubble destruction. Subsequently, 20 cardiac cycles were recorded with low MI imaging at a frame rate of 18 Hz to allow contrast replenishment in the myocardium. All data were stored for offline analysis.

### Evaluation of autonomic function

All subjects underwent a set of standard autonomic function tests to screen for cardiovascular autonomic neuropathy. First, subjects are placed in the supine position and heart rate (R – R intervals) was recorded for 5 min during spontaneous breathing. Subsequently, autonomically induced variations in R – R intervals to changes in blood pressure were recorded during one minute of deep breathing, during the Valsalva manoeuvre (Valsalva ratio) and quick stand test. R – R intervals and blood pressure were continuously recorded using a non-invasive continuous finger arterial blood pressure measurement device with a sample rate of 200 Hz (Nexfin HD, BMEYE, Edwards Lifesciences, The Netherlands). Data was stored on a personal computer for further analysis using free available software (Kubios HRV version 2.0, University of Kuopio, Finland and Beatscope, BMEYE, Edwards Lifesciences, The Netherlands). During spontaneous breathing over 5 minutes, heart rate variability (HRV) was assessed by spectral analysis using fast Fourier transformation. This method divides the overall variability of a signal into its composing frequencies and provides insight into what extent a frequency contributes to the overall variability. The power spectrum of HRV consists of three peaks: the very-low-frequency band (<0.04 Hz), the low-frequency band (0.04 – 0.12 Hz) and the high-frequency band (0.12 – 0.40 Hz). The very-low-frequency fluctuations mediated primarily by the sympathetic system, the low-frequency fluctuations by both the sympathetic and parasympathetic system and the high-frequency fluctuations are under parasympathetic control.

From the recordings during deep breathing, maximum and minimum R – R intervals were extracted and expressed as a ratio, which in healthy subjects should be larger than 1.17. For the Valsalva manoeuvre, subjects exhaled forcibly through a manometer against a pressure of 40 mmHg for 15 s. The ratio of R – R intervals was calculated, being > 1.21 in healthy subjects. Assessment of heart rate response during quick standing, R – R intervals were measured at 15 and 30 beats after standing. In healthy subjects, the ratio of the longest R – R interval to the shortest R – R interval is > 1.04. To assess changes in blood pressure during standing, systolic blood pressure was measured in the resting, supine subject. Two minutes after rapid standing, the measurement was repeated. In healthy subjects the fall in systolic blood pressure after the test should be less than 10 mmHg. Cardiac autonomic neuropathy is defined as the presence of 3 or more abnormal test results among these 7 tests [19].

### Anaesthesia

Heart rate, blood pressure and oxygen saturation were monitored throughout the protocol. All patients received midazolam 0.02 mg kg^-1^ intravenously. Anaesthesia was induced by sevoflurane inhalation (AbbVie, Hoofddorp, The Netherlands) and maintained at an age-adjusted end-tidal concentration of 1.0 minimum alveolar concentration. A laryngeal mask airway was inserted and patients continued to breathe spontaneously without positive airway pressure during the study period. The surgical procedure started after MBF measurements were completed.

### Analysis of contrast echocardiography

Semiautomated software providing manual region of interest (ROI) tracking, visualisation of perfusion calculations and dataset handling was used (PerfusionFitter, Department of Cardiology, Bern University Hospital). ROIs were drawn in end-systolic frames in the mid-inferoseptal and mid-anterolateral wall (apical 4-chamber view); in the mid-inferior and mid-anterior wall (apical 2-chamber view) and in the mid-inferolateral and mid-anteroseptal wall (apical 3-chamber view) of the myocardium. Using the volumetric model by Vogel *et al.*, myocardial blood flow was quantified in ml min^-1^ g^-1^ from the underlying microvascular parameters: (1) the relative myocardial blood volume, which represents the blood volume in the capillary system, and (2) the capillary exchange rate, which provides an estimate of the exchange rate of erythrocytes within the region of interest [20]. Changes in blood pressure and heart rate during the interventions were monitored at regular intervals. Additional calculations were made to provide an estimate of myocardial oxygen consumption (rate-pressure product [RPP]; heart rate x systolic blood pressure) and coronary vascular resistance (CVR; dividing mean arterial blood pressure by MBF).

### Statistics

All data are presented as mean ± SD unless indicated otherwise. Baseline characteristics between controls and diabetics were compared using a Mann–Whitney U test. A Wilcoxon signed-rank test was used for within group comparisons of myocardial blood flow results (baseline versus sevoflurane). Changes in myocardial blood flow responses before and during sevoflurane administration were compared between controls and diabetics using a Mann–Whitney U test. Descriptive statistics were provided for hemodynamic data. A P < 0.05 was considered as statistically significant.

## Results

### Baseline characteristics

Table 1 shows baseline characteristics of the study population. Eight cardiovascular healthy males and six diabetic males were included in this pilot study. Type 2 diabetic patients were older than control subjects. No statistical difference in BMI is observed between groups. Fasting glucose and HbA1c-levels were higher in patients with type 2 diabetes. Further, total and LDL-cholesterol levels were lower in diabetics.

**Table 1:** Demographic data of study population

	**Controls (N = 8)**	**Diabetics (N = 6)**
Age, years (range)	44 (28 – 59)	57 (42 – 71)*
BMI, kg/m^2^	25.7 ± 3.4	30.7 ± 9.4
Duration of diabetes (year)	-	7.7 ± 5
Fasting blood glucose (mmol/l)	4.9 ± 0.4	8.5 ± 1.5*
HbA1c (%)	5.4 ± 0.2	6.8 ± 0.8*
Total cholesterol (mmol/l)	5.1 ± 0.8	3.7 ± 0.3*
LDL cholesterol (mmol/l)	3.1 ± 0.7	1.8 ± 0.3*
HDL cholesterol (mmol/l)	1.3 ± 0.5	1.2 ± 0.4
Triglycerides (mmol/l)	1.4 ± 0.8	2.5 ± 2.9
Creatinine (μmol/l)	83 ± 12	69 ± 10
Urea (mmol/l)	5.5 ± 0.8	5.0 ± 0.9
Oral antidiabetic therapy (nr of patients)		
Metformin		4
Glimepiride		3
Exenatide		1
Gliclazide		1
Liraglutide		1
Pioglitazone		1

BMI: body mass index; LDL: low-density lipoprotein; HDL: high-density lipoprotein. Data are represented as mean ± SD unless indicated otherwise; Mann–Whitney U test, * p < 0.05.

### Autonomic function

Clinical assessment of autonomic function in the control group revealed no abnormal responses to the tests (Table 2). In the diabetic group, one patient was identified with autonomic dysfunction based on three abnormal test results (heart rate response to deep breathing and standing, blood pressure response to standing). One patient had two abnormal responses (power spectrum in the low-frequency band and heart rate response to deep breathing), indicating borderline autonomic dysfunction.

**Table 2:** Results of autonomic function tests under standardised conditions

	**Controls (N = 8)**	**Diabetics (N = 6)**
**HRV Very low power spectrum (ms**^**2**^**)**	906 (305–2693 [160–4835])	1031 (410–8501 [63–8573])
*< 0.04 Hz*		
**HRV Low power spectrum (ms**^**2**^**)**	844 (275–1513 [190–1662])	526 (53–5427 [24–8712])
*0.04 – 0.12 Hz*		
**HRV High power spectrum (ms**^**2**^**)**	1202 (214–1513 [66–2711])	507 (74–2185 [37–4112])
*0.12 – 0.40 Hz*		
**Deep breathing**	1.25 ± 0.08	1.19 ± 0.11
*Heart rate response ratio*		
**Valsalva maneuver**	1.75 ± 0.27	1.56 ± 0.23
*Heart rate response ratio*		
**Quick stand**	1.56 ± 0.29	1.31 ± 0.31
*Heart rate response ratio*		
**Quick stand (mmHg)**	1 ± 9	-3 ± 11
*Blood pressure response*		
**Presence of autonomic dysfunction**	0/8	1/6
*Number of patients*		

Data are presented as median (IQR [range]) or mean ± SD.

### Haemodynamics

At baseline, heart rate and RPP increased in both groups during adenosine infusion (Table 3). These responses were blunted in both groups during adenosine-induced hyperaemia under sevoflurane anaesthesia. The CPT at baseline increased SBP and RPP in diabetics and controls. Under sevoflurane anaesthesia, the CPT decreased SBP and RPP in healthy controls, while heart rate and RPP were decreased in diabetics.

**Table 3:** Descriptive statistics of haemodynamic parameters at baseline and during sevoflurane anaesthesia

	**Controls (N = 8)**		**Diabetics (N = 6)**	
	***Baseline***	***Sevoflurane***	***Baseline***	***Sevoflurane***
**Rest**				
*HR*	62 ± 8	66 ± 12	71 ± 14	75 ± 16
*SBP*	111 ± 10	96 ± 17	128 ± 17	104 ± 16
*DBP*	76 ± 8	53 ± 12	76 ± 13	58 ± 20
*RPP*	6812 ± 816	6338 ± 1517	9209 ± 2814	7660 ± 1482
*CVR*	98 ± 27	76 ± 25	84 ± 12	106 ± 70
**Hyperemia**				
*HR*	87 ± 15	78 ± 11	90 ± 7	77 ± 20
*SBP*	120 ± 14	80 ± 9	131 ± 24	90 ± 18
*DBP*	76 ± 5	44 ± 6	77 ± 17	49 ± 20
*RPP*	10450 ± 2225	6207 ± 1068	11890 ± 2840	6612 ± 1736
*CVR*	29 ± 5	26 ± 3	36 ± 12	66 ± 53
**CPT**				
*HR*	67 ± 6	65 ± 13	78 ± 11	69 ± 13
*SBP*	131 ± 21	81 ± 6	152 ± 21	97 ± 9
*DBP*	80 ± 10	40 ± 9	88 ± 16	50 ± 12
*RPP*	8772 ± 1409	5208 ± 794	11920 ± 3082	6680 ± 999
*CVR*	70 ± 24	45 ± 6	95 ± 31	56 ± 49

Data are presented as mean ± SD, descriptive statistics. CPT = cold pressor test; CVR = coronary vascular resistance (mmHg min g ml^-1^); DBP = diastolic blood pressure (mmHg); HR = heart rate (min^-1^); RPP = rate pressure product (min^-1^ mmHg); SBP = systolic blood pressure (mmHg).

### Myocardial blood flow measurements

**Figure 1:**
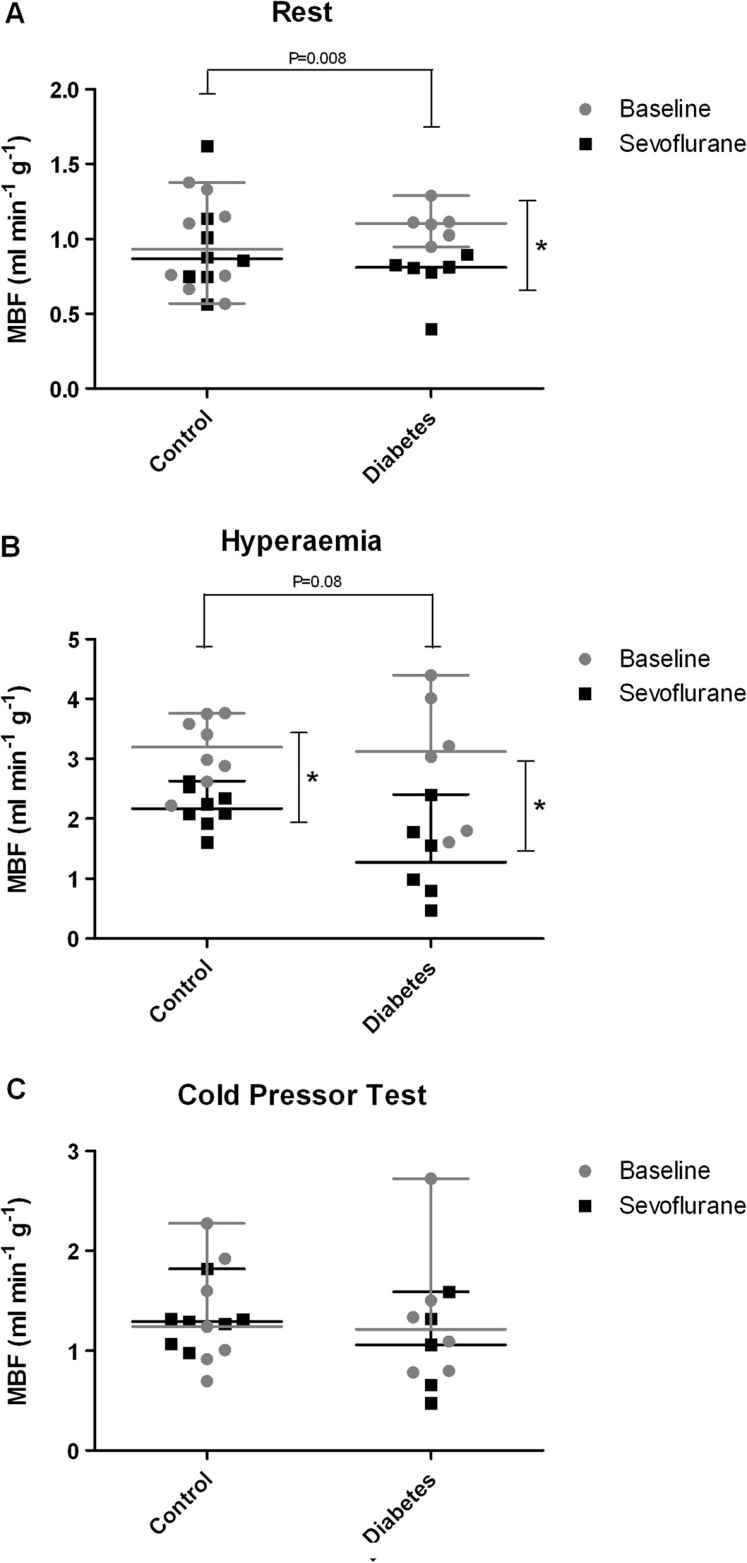
**Myocardial blood flow measured with contrast echocardiography.** Myocardial blood flow at rest **(A)** and after adenosine infusion **(B)** and cold pressor testing **(C)** in healthy controls and diabetic patients. Dot plots indicate median ± range. CPT = cold pressor test; MBF = myocardial blood flow. A Wilcoxon signed-rank test was used for within group comparisons of myocardial blood flow results (baseline versus sevoflurane). Changes in myocardial blood flow responses before and during sevoflurane administration were compared between controls and diabetics using a Mann–Whitney U test.

Sevoflurane anaesthesia decreased MBF in diabetics but not in controls (P = 0.03). At baseline, adenosine-induced hyperaemia increased MBF in both groups compared to resting values. However, when adenosine was combined with sevoflurane anaesthesia, the MBF was significantly lower in both controls and diabetics when compared to baseline conditions (Figure 1). Differences in MBF response to adenosine before and after sevoflurane administration were larger in diabetic patients, this was however not statistically significant in this pilot group (P = 0.08). Myocardial blood flow parameters after the cold pressor test were not different between groups at baseline and during sevoflurane exposure.

## Discussion

In this pilot study, we show that sevoflurane anaesthesia impairs resting myocardial blood flow in type 2 diabetic patients, while it preserves myocardial blood flow in healthy controls. Furthermore, we observed a decrease in hyperaemic MBF (endothelium-independent vasodilation) during sevoflurane anaesthesia in both groups, with a trend towards a larger decrease in diabetic patients. Sympathetic stimulation under sevoflurane did not lead to changes in MBF in controls or diabetics when compared to baseline (endothelium-dependent vasodilation). These findings suggest different myocardial responses to sevoflurane anaesthesia in type 2 diabetic patients compared to healthy controls.

Type 2 diabetes mellitus leads to endothelial dysfunction of the coronary circulation due to reduced availability of nitric oxide, accumulation of glycation end products in the arterial wall, impaired insulin signalling, inflammation and other still unknown mechanisms [7,21]. Picchi *et al.* showed that diabetic coronary dysfunction is clinically characterised by increased basal MBF and similar hyperaemic MBF compared to healthy patients [22]. In contrast, Di Carli *et al.* reported similar resting MBF and lower hyperaemic MBF in diabetics compared to controls [9]. The additional influence of sevoflurane anaesthesia on MBF and endothelial function in diabetic patients is largely unknown. Our type 2 diabetic patients had a lower resting MBF when compared to baseline and this sevoflurane effect on MBF was different from healthy controls. Interestingly, Cosyns *et al.* reported no difference in resting MBF in streptozotocin-induced type 1 diabetic rats compared to controls rats under thiopental anaesthesia [23]. No studies on sevoflurane anaesthesia are available for comparison with our data.

Sevoflurane anaesthesia decreased MBF during adenosine-induced hyperaemia in both groups when compared to baseline conditions. This was in accordance with our previous study in healthy volunteers [17]. Also, diabetics tended to have a larger decrease in MBF than healthy controls. Cosyns *et al.*, showing higher hyperaemic MBF in controls than in diabetic rats, confirm the latter observation [23]. Underlying mechanisms for this observation may include changes in perfusion pressure and myocardial oxygen demand, decreased capillary recruitment in diabetic myocardium and direct effect of sevoflurane on endothelial cells [24].

Endothelial-dependent vasodilation by the CPT during sevoflurane anaesthesia was not different from baseline in both controls and diabetic patients. It has been shown that the MBF response to sympathetic stimulation is primarily dependent on the integrity of the autonomic nervous system and not on hemodynamic changes or circulating catecholamine levels [25]. Also, it was shown by the same investigators that impaired MBF responses to endothelium-dependent vasodilation in type 2 diabetic patients were related to the degree of cardiac autonomic dysfunction [9]. In our population, only one out of six diabetic patients had clinical evidence of autonomic dysfunction, which hampers evaluation of MBF responses in this subpopulation under sevoflurane anaesthesia. Future study designs should also be aimed at type 2 diabetics with cardiac autonomic dysfunction.

For this study sevoflurane anaesthesia was used since it is widely available and allows both induction and maintenance of anaesthesia. Volatile anaesthetics are thought to possess cardioprotective properties although experimental evidence suggests that these properties are reduced in type 2 diabetes [26]. Interestingly, a recent study reported that the widely used intravenous anaesthetic propofol attenuates hyperglycaemia-induced upregulation of endothelial adhesion molecules expression and mononuclear-endothelial adhesion [27]. This implicates that propofol inhibits pathological leukocytes-endothelial adhesion and may subsequently prevent endothelial dysfunction and injury. Furthermore, comparing between sevoflurane and propofol, it has been reported that sevoflurane better preserved right ventricular function than propofol in patients receiving esophagectomy. However, propofol was better in improving oxygenation and shunt-fraction during one-lung ventilation [28]. The results of these studies suggest that the choice of anaesthetic may be of interest in certain patient categories. Specifically in type 2 diabetics, influence of the two most widely used agents on myocardial perfusion and function should be further investigated.

All type 2 diabetic patients in this study received oral antidiabetic therapy (see Table 1), which are known to exert cardiovascular effects and may affect myocardial blood flow. Previous studies reported cardioprotective effects exerted by metformin mediated by protein kinase B, adenosine monophosphate-activated protein kinase and upregulation of insulin signalling pathways [29,30]. Also, metformin improved endothelium-dependent vasodilation in both nondiabetic and diabetic subjects [31,32]. In isolated rat hearts, sulfonylurea derivatives reduced myocardial perfusion under ischemic conditions, while perfusion under normal conditions was unaffected [33,34]. In humans, these oral diabetics deteriorated both resting and hyperaemic myocardial blood flow [35]. In type 2 diabetic patients, exenatide increased myocardial blood flow, while treatment with peroxisome proliferator-activated gamma agonists did not affect myocardial perfusion [35-37]. Whether and how antidiabetic therapy influences the results of this study cannot be concluded.

The small number of patients studied limits interpretation of the present study results. However, pilot studies are valuable and necessary to enable power calculation for future studies. Ideally, in subsequent studies age-matched controls should be used for comparison with diabetic subjects. The age difference between healthy controls and diabetic subjects in this study could be a potential confounder.

Already in this small study population we showed that sevoflurane reduces resting MBF in diabetic patients compared to healthy controls. Hyperaemic MBF decreased during sevoflurane anaesthesia in both groups. Next, an adequately powered study should be undertaken; for hyperaemia a sample size of 21 in each group will have 80% power to detect a significant difference using a two-group t-test with a 0.05 two-sided significance level. For cold pressor testing, a sample size of 92 in each group will have 80% power to detect a significant difference using a two-group t-test with a 0.05 two-sided significance level. Furthermore, assessment of effects of hypo- and hyperinsulinemia on vascular reactivity via nitric oxide and insulin signaling proteins such as Akt and endothelial nitric oxide synthase combined with altered plasma levels of endothelin-1, asymmetric dimethylarginine and nitric oxide may elucidate mechanisms behind sevoflurane-induced MBF changes in diabetic patients [38-41].

## Conclusions

In conclusion, these pilot data suggest that sevoflurane anaesthesia decreases resting myocardial blood flow in type 2 diabetic patients compared to healthy controls. Further, we observed a trend towards a lower endothelium-independent vasodilation capacity in diabetic patients under sevoflurane anaesthesia. These data provide preliminary insight into myocardial responses in type 2 diabetic patients under general anaesthesia.

## Abbreviations

BMI: Body mass index; CPT: Cold pressor test; CVR: Coronary vascular resistance; DBP: Diastolic blood pressure; HR: Heart rate; HRV: Heart rate variability; HDL: High-density lipoprotein cholesterol; LDL: Low-density lipoprotein cholesterol; MBF: Myocardial blood flow; MCE: Myocardial contrast echocardiography; MI: Mechanical index; ROI: Region of interest; RPP: Rate-pressure product; SBP: Systolic blood pressure.

## Competing interests

The authors declare that they have no competing interests.

## Authors’ contributions

CSEB participated in performing the study, data analysis, statistics and writing of the manuscript. CEvdB supported the data analysis and writing of the manuscript. SAL participated in the design of the study and reviewed/edited the manuscript. CB participated in the design of the study, data analysis and reviewed/edited the manuscript. RAB participated in the design of the study, data collection and reviewed/edited the manuscript. All authors read and approved the final manuscript.
